# Skin appendage-derived stem cells: cell biology and potential for wound repair

**DOI:** 10.1186/s41038-016-0064-6

**Published:** 2016-10-26

**Authors:** Jiangfan Xie, Bin Yao, Yutong Han, Sha Huang, Xiaobing Fu

**Affiliations:** 1 Key Laboratory of Tissue Repair and Regeneration of PLA, and Beijing Key Research Laboratory of Skin Injury, Repair and Regeneration, First Hospital Affiliated to General Hospital of PLA, 51 Fu Cheng Road, Beijing, 100048 People’s Republic of China; 2 School of Medicine, Nankai University, Tianjin, 300052 People’s Republic of China; 3 Graduate School of the Second Teaching Hospital of Zhengzhou University, Zhengzhou, 450000 People’s Republic of China; 4 Wound Healing and Cell Biology Laboratory, Institute of Basic Medical Sciences, General Hospital of PLA, Beijing, 100853 People’s Republic of China

**Keywords:** Skin appendages, Stem cells, Cell biology, Wound healing

## Abstract

Stem cells residing in the epidermis and skin appendages are imperative for skin homeostasis and regeneration. These stem cells also participate in the repair of the epidermis after injuries, inducing restoration of tissue integrity and function of damaged tissue. Unlike epidermis-derived stem cells, comprehensive knowledge about skin appendage-derived stem cells remains limited. In this review, we summarize the current knowledge of skin appendage-derived stem cells, including their fundamental characteristics, their preferentially expressed biomarkers, and their potential contribution involved in wound repair. Finally, we will also discuss current strategies, future applications, and limitations of these stem cells, attempting to provide some perspectives on optimizing the available therapy in cutaneous repair and regeneration.

## Background

Skin as a barrier for resisting external invasion is distributed to every part of the body, which concludes the epidermis and dermis [1]. Morphologically, the epidermis is the structure in the skin’s outermost layer, and it together with its derivative appendages protects the organism from the outside, as well as regulates the body temperature and homeostasis [2]. Like other organs, there are some related stem cells in the skin and its derived appendages, which have the capacity to maintain homeostasis, self-renew tissue, and contribute to wound healing [3]. Skin wound healing is a highly organized and coordinated series of processes that leads to the restoration of tissue integrity and function [3]. A slice of factors can cause an interruption in wound healing including systemic and local effects. Systemic effects include compromised nutritional, immune status, diabetes, and advanced age. Local factors include tissue hypoxia, ischemia, foreign bodies, maceration of tissue, exudates, and infection [4]. Several therapies have emerged for chronic wounds, with different degrees of success [4, 5]. However, the report of autoallergic repair by skin appendage-derived progenitor/stem cells remains limited.

This review aimed primarily to introduce the skin appendage-derived progenitor/stem cells, including their characteristics, functions, therapeutic potentials, and limitations as therapeutic tools for wound healing. In the following sections, we defined skin appendage-derived progenitor/stem cells and summarized some of the biomarkers used for their identification based on reported researches, and discussed their potentials in wound healing and limitations.

## Review

### Skin appendage-derived progenitor/stem cells

**Fig. 1:**
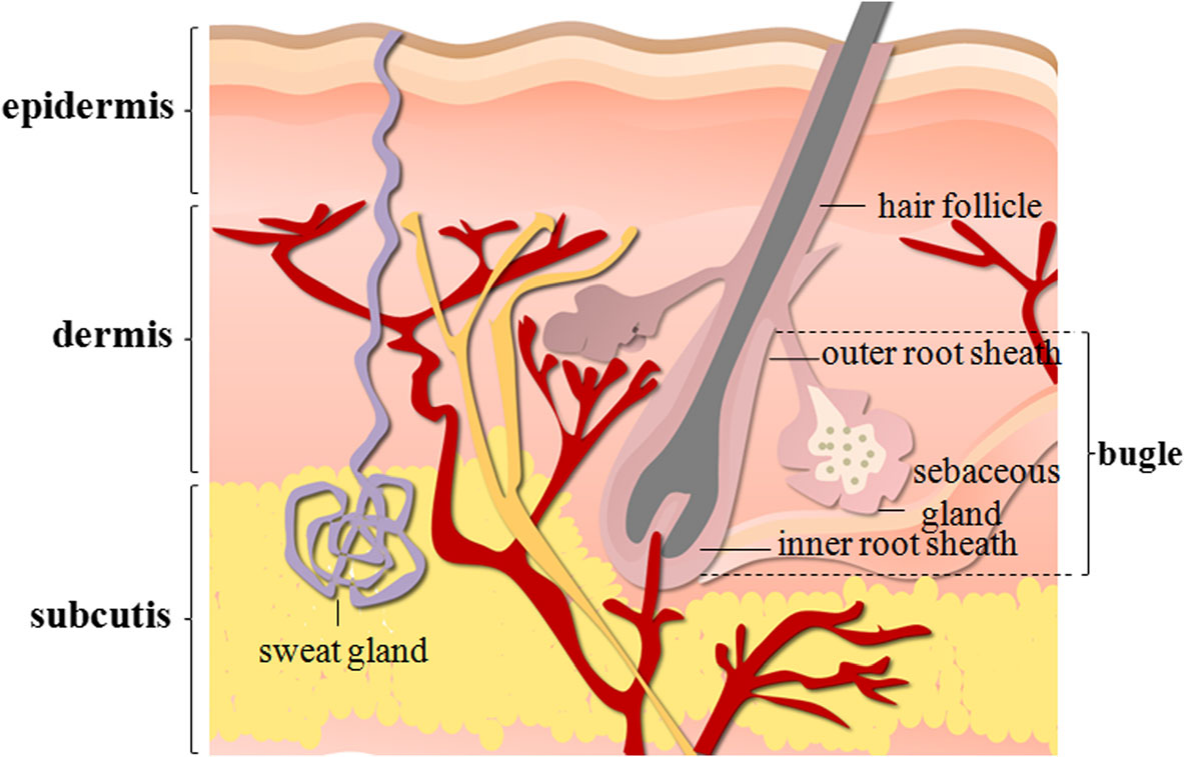
The schematic diagram of full-thickness skin

Skin appendages develop during the embryonic period following a precise spatiotemporal pattern involving complex interactions between the cells from primitive epidermis, and ectodermic origin, as well as the underlying mesenchymal cells from mesodermic origin (Fig. 1). Epithelial stem cells rely heavily on quiescence as a major stem cell characteristic, which is due to “label-retaining cell” methods for detecting quiescent cells in the epidermis from the pioneering work of Bickenbach and Mackenzie [6].

#### Eccrine sweat gland progenitor/stem cells

Sweat gland as a crucial skin appendage widely exists in the human skin surface, while only distributed on the palm of mice, which plays a pivotal role in the process of temperature regulation and homeostasis [7, 8]. In human embryos, sweat gland buds begin to emerge on the palms and soles at 12–13 weeks and on the rest of the body at 20 weeks [9]. At 22 gestational weeks of human, myoepithelial cells and luminal cells in the secretory portion can be detected. In mice, sweat gland buds first appear at embryo 16.5 days. It completes maturation at day 14 after birth and fully function at day 21 after birth [9, 10]. In the process of development, sweat gland germs grow down progressively into the dermis to form a duct, ending in a secretary coil, and play their function basing on the body itself and the changes in the external temperature. The mature sweat glands contain the portion of duct and secretary coil.

Knowledge of the eccrine sweat gland is relatively unified, which is differentiated from epithelial-derived stem cells and its progenitors. Recently, Lu et al. and Van Keymeulen et al. found the existence of two populations of stem cells in the sweat ducts of postnatal mice and four populations in the paw skin of adult mice [11, 12]. Sweat glands begin to develop as a multipotent sweat bud progenitor (K14^+^) during fetal life. After stratification, K18 expression is increased and K14 expression reduced, which generates a transient but proliferative suprabasal layer of progenitors (K14^low^/K18^+^) [11]. Finally, these basal and suprabasal ductal progenitors continue to differentiate and migrate outwards to form myoepithelial and luminal progenitor cells successively [11, 13]. In adult paw skin, both luminal progenitors and myoepithelial progenitors at sweat ducts and secretory portion make a significant effect in homeostatic turnover, and each followed distinct basal → suprabasal differentiation programs [11, 14].

Whether in human or in mice, these cells of sweat glands express a number of characterized markers. Luminal cells of a mature sweat gland express K8 and K18 [15]. NKAα, ATP1a1, and K19 are expressed in the gland portion of a mature sweat gland [16, 17]. K14 and K5 are found in the myoepithelial cells [10]. K10 is expressed positively in the duct portion of sweat gland cells [10]. Moreover, we can identify its stemness by Nestin, CD9, CD29, CD44, and CD81 expressed in the sweat glands [17] (Table 1).

**Table 1:** The expression patterns of skin appendage markers

Skin appendage	Cells	Markers
Sweat gland	Mature luminal cells	K8, K18
	Mature gland cells	NKAα, ATP1a1, K19
	Mature myoepithelial cells	K14, K5
	Mature duct cells	K10
	Stemness	Nestin, CD9, CD29, CD44, CD81
	Sweat bud progenitor	K14 → K14low/K18+
Hair follicle	Bugle cells	K15, CD34, SRY box 9
	Stem cells reside in the upper isthmus	(Plet1)/MTS24, Lrig1
	Stem cells reside in the lower isthmus	Lgr6 and Gli1
	Epidermal stem cells in the HF bulge	GATA3, BMPR1a, ID2, ID4, Wnt, β-catenin
	Human hair follicle bulge	K15, PHLDA1, CD200, K19
Sebaceous gland	Sebaceous gland progenitors	Blimp1, K5, K14

*Plet1* placenta transcript 1, *Gli1* glioma-associated oncogene homolog 1, *GATA3* GATA binding protein 3, *BMPR1a* bone morphogenetic protein receptor1a, *ID2* DNA-binding protein 2, *PHLDA1* pleckstrin homology-like domain, family A member 1, *CD200* cluster of differentiation 200

#### Hair follicle progenitor/stem cells

The morphology of hair follicle begins at the early period of embryo and signals from the epithelium inducing the formation of dermal condensates [2]. The first wave of hair placodes of mouse starts by the mesenchymal–epithelial interactions at embryonic day (E) 14.5 [2]. Once initiated, these placodes undergo proliferation and downgrowth to form first hair germs at E15.5 and hair pegs at E16.5–17.5. Then, the inner root sheath (IRS) provides the channel for the emerging hair (E18.5) [2]. At birth, the mature hairs begin to break the skin surface. After the first postnatal week, the full maturation hair follicle is formed [2, 18]. In general, there are four waves of follicle morphogenesis during the developmental process of hair follicle, with the large primary guard hairs forming first (E14.5) and followed by the bulk of the hair coat follicles starting to form [19].

Hair follicle stem cells are likely persistent throughout the lifetime of the organism [20]. There are two main subpopulations of stem cells in hair follicles: the one under the bulge gives rise to the hair shaft and IRS and the other residing in the bulge region gives rise to the basal outer root sheath (ORS) keratinocytes [3]. Owing to the characteristic of slow cycling, quiescent nature, clonogenic capability, and the expression of a subset of markers, the bugle region has been known as the most well-defined stem cell niche in the skin [20, 21]. The reservoir for follicle stem cells is formed during the process of each new anagen forming [2, 12]. Moreover, some researchers have found that the offspring of bugle cell can form sebaceous glands and epidermis as well as new hair follicles [22]. It suggests that follicle stem cells only contribute to hair follicle homeostasis, whereas they are not involved in interfollicular epidermis regeneration [23].

In mice, the bugle cells of hair follicle express K15, CD34, and SRY box 9 [24–27]. Stem cells reside in the upper isthmus/junctional zone region can express placenta transcript 1 (Plet1)/MTS24 and Lrig1. Lgr6- and glioma-associated oncogene homologue 1 (Gli1) are expressed in the cells which are located in the lower isthmus [28, 29]. Furthermore, GATA binding protein 3 (GATA3), bone morphogenetic protein receptor1a (BMPR1a), the inhibitors of DNA-binding protein 2 and 4 (ID2, ID4), and Wnt and β-catenin also can regulate epidermal stem cells and their fate in the hair follicle (HE) bulge [30]. K15, pleckstrin homology-like domain, family A member 1 (PHLDA1), cluster of differentiation 200 (CD200), and K19-positive cells can be found in the human hair follicle bulge too [31, 32] (Table 1).

#### Sebaceous gland progenitor/stem cells

Sebaceous gland as an integral philosebaceous unit resides above the bugle and under the hair shaft orifice at the skin surface and secretes sebum to lubricate the skin and keeps the waterproof property of hair in mammals [2]. In human embryos, sebaceous glands begin to emerge approximately at 13–14 weeks [7, 33]. In mice, sebaceous glands develop around the end of embryogenesis and fully function after birth [7, 33]. At birth, the sebaceous gland precursor cells form the upper segment of the root. Moreover, Horsley et al. have found that a small population of cells near or at the base of sebaceous glands maintained stemness [34]. Furthermore, sebaceous gland progenitors of normal mice express Blimp-1 [34]. And the Blimp1-positive sebaceous gland cells also express K5/K14 [3] (Table 1).

#### Other skin appendage-derived progenitor/stem cells

Except for eccrine sweat glands, hair follicle, and sebaceous glands, there are other appendages in the epidermis including melanocytes. Melanocyte stem cells mainly reside in the hair follicle bulge region and hair germ, which express dopachrome tautomerase, Sox10, and paired box 3 [35]. These stem cells form mature melanocytes in the hair follicle bulb. Several studies have demonstrated that cell–cell interaction in epidermal stem cells via Wnt signaling, TGF-β, notch signaling, nuclear factor I/B (NFIB), and Col17a1 can regulate melanocyte stem cells [36, 37]. Notably, melanocyte stem cells could migrate upwards to the interfollicular epidermis (IFE) and differentiate into functional epidermal melanocytes contributing to the wound healing [38, 39].

### Potential of skin appendage-derived progenitor/stem cells for wound repair

Skin as the first barrier of our body resists the encroachment. Thus the rapid repair is crucial to maintain its function. Previous studies using lineage tracing and mouse models indicated that stem cells of both the epidermis and its appendages contribute to skin wound healing.

#### Eccrine sweat gland progenitor/stem cells in wound healing

To search decisional assessment of the source of stem cells in wound healing process, Lu et al. marked four stem cell populations and established new models [11]. They revealed that eccrine sweat ductal cells migrated and repaired an epidermal scratch wound in 3 days and restored the intraepidermal sweat ducts in 14 days. Only sweat ductal basal progenitors responded and proliferated to repair the duct orifice extending throughout the skin surface, indicating that sweat duct is the growth center after epidermis injury. In contrast, luminal and myoepithelial cells from the secretory section of sweat glands functioned as unipotent progenitors during glandular repair. These findings provided evidence that sweat gland stem cells might possess significant potential for clinical applications including extensive burns and massive skin loss [40]. Although ductal stem cells seemed accused on repairing the sweat duct orifice, the epidermal stem cells adjacent to the wound showed more athletic in epidermal repair, which indicated that both ductal and epidermal progenitors participate in epidermal wound repair [41]. Our research group has explored a series of strategies to facilitate sweat gland regeneration on wound healing. MSCs exist in bone marrow stroma, which are relatively slight damged during isolation and easily activated in large area burn and trauma. Moreover, others’ pre-researches and ours have proved that MSCs are directly involved in the whole process of repairing and regeneration of skin damage, including sweat gland repair [42, 43]. Based on these backgrounds, the studies of our laboratory have confirmed that MSCs cultured in vitro could differentiate into cells with the phenotype of sweat gland cells. If these MSCs were transplanted into the wound, they could be transformed into vascular endothelial cells, epidermal cells, sebaceous duct cells, and sweat gland cells in granulation tissue [43, 44]. Meanwhile, Huang et al. have showed that the epidermal stem cells could be induced into sweat glands like cells with sweat gland function under 3D-printed microenvironment [45].

#### Hair follicle and sebaceous gland progenitor/stem cells in wound healing

Both clinical and experimental evidence suggest that hair follicle and sebaceous gland progenitor cells contribute to the re-epithelialization of wounds [23]. Some researchers have shown that cells from hair follicles and IFE would migrate to the wound portion in full-thickness [23]. Fate-mapping experiments demonstrated that hair follicle stem cells only exist in wounds [23]. In response to skin injury, Gli1+, Lrig1+, Lrg5+, and MT24+ cells become activated and contribute towards IFE repair, suggesting the plasticity of epidermal stem cells [46, 47]. Jimenez et al. evaluated the feasibility and potential healing capacity of autologous scalp follicular grafts transplanted into the wound by detecting the epithelialization, neovascularization, and dermal reorganization of chronic leg ulcers in ten patients after 18 weeks, demonstrating hair follicle grafting as a promising therapeutic strategy for non-healing chronic wounds [48]. Moreover, other group reported that hair follicle progenitors were largely replaced by epidermal progeny in wound healing [49]. These findings suggest that both IFE and hair follicle stem cells contribute to wound healing [50, 51].

Some researchers confirmed that the stem cells of the root sheath and bugle can repair not only the hair follicle but also the sebaceous gland on the process of wound healing [2, 23]. Sweat gland stem cells can renew themselves and repair epidermis on wound healing [9]. Although previous studies revealed that stem cells of these skin appendages contribute to skin wound repair, the interaction among these appendage-derived progenitor/stem cells on wound healing is still unclear [23, 47]. Because there are more sweat glands than hair follicles in the majority area of human skin, it has been hypothesized that sweat gland would assist other skin appendages on wound healing, and the concrete mechanism and evidence needed to further explore.

### Limits of skin appendage-derived progenitor/stem cell application

Skin appendages or appendage-derived stem cells have thrilled so many biomedical researchers over the last 10 years. PubMed, in 2015, collected over 867 references for “skin appendage” and more than 7725 for “skin appendage-derived stem cells.” Part of them are comprehensive recent reviews on skin appendages and skin appendage-derived stem cells. However, studies treating wound healing with these stem cells are rare. We summarized limitations and difficulties from different points of view about skin appendage-derived stem cells on wound healing.

The cell source is certainly limited. Lu et al. found four types stem cells of the eccrine sweat gland at 2012 [11], while the specific methods of isolating stem cells of the eccrine sweat glands could only get limited numbers. Other investigators also explored knowledge of hair follicle- and sebaceous gland-derived stem cells, but the isolating methods are absent. Because of the special period and portion and limited quantity of appendage-derived stem cells, they are difficult to isolate and purify. Moreover, although there are many researches of skin appendage-derived stem cells on wound healing recently, the mechanisms at molecular and cellar level are not distinct.

Mice were the most commonly used animal mode for researches of skin appendage-derived stem cells, but it is difficult to establish the identical wound. We usually established chronic non-healing wounds mice model through burns or diabetes mellitus wounds, while the changes were not only involved in wound region. For example, a burn patient would not only develop a series of metabolic changes in wound site but also have significant change of homeostasis of the whole body, such as water and salt balance. In chronic non-healing wounds caused by diabetes in patients usually with elder age, they might have diseases including hypertension, hyperlipidemia, and renal involvement generally. The complex symptom is difficult to completely reproduce in animal models. Stem cell therapy is usually directly injected to the specific site or intravenous injection, but the exact number of cells that arrived at the wound site and the interaction time could not be determined. Hence, a suitable approach to study the role of stem cells in the wound site is in urgent need. Not to mention, there are some problems that blocked the transformation of basic experiments to clinical trials.

## Conclusions

In this review, we have discussed the characteristics and functions of eccrine sweat gland-, hair follicle-, and sebaceous gland-derived progenitor/stem cells as well as summarized the relationship, potential, and limits of these stem cells on wound healing. We mainly elucidated the roles of skin appendage-derived progenitor/stem cells in wound healing, associated with their development and molecular mechanisms. Chronic non-healing wounds are common and need to be paid attention. Skin appendage-derived progenitor/stem cells could reduce the immune response and promote repair. To figure out the comprehensive cellular interactions and stem cell behavior would facilitate wound healing. With the knowledge of skin appendage-derived progenitor/stem cells recently showed that choosing the right stem cell type for fully functional skin regeneration in vivo is extremely important. Furthermore, identifying and isolating pure skin appendage-derived progenitor/stem cell populations, exploring the underlying mechanisms, and optimizing protocols for cell delivering to appropriate portion need to be further explored. Moreover, we need more clinical trials to further explore the long-term effects with these cells and ultimately provide safer and more effective therapies for future clinical applications.
